# Drug-Induced Sleep Endoscopy: Clinical Application and Surgical Outcomes

**DOI:** 10.3390/healthcare7030100

**Published:** 2019-08-25

**Authors:** Andrea De Vito, Giovanni Cammaroto, Khai Beng Chong, Marina Carrasco-Llatas, Claudio Vicini

**Affiliations:** 1 Head and Neck Department, ENT unit, Morgagni-Pierantoni Hospital, AUSL of Romagna, 47121 Forlì, Italy; 2 Department of Otorhinolaryngology, Tan Tock Seng Hospital, Singapore 308433, Singapore; 3 ENT Department, Hospital Universitario Doctor Peset, 46017 Valencia, Spain

**Keywords:** drug-induced sleep endoscopy, DISE, sedation

## Abstract

The visualization of the level and pattern of apnea and hypopnea events is of pivotal importance in the diagnosis and therapeutic decision-making for sleep-disordered breathing (SDB). There are numerous techniques available to assess upper airway obstruction, which include imaging, acoustic analysis, pressure transducer recording, and endoscopic evaluation. Drug-induced sleep endoscopy (DISE) is a diagnostic tool that allows the dynamic, three-dimensional evaluation of the patterns of vibration and collapse of the upper airway of SDB patients. DISE may change the initial surgical planning in a high percentage of cases. A universally accepted and methodologically standardized DISE could provide significant insight into its role to improve surgical outcomes. However, up to now the ideal DISE protocol remains an open question.

## 1. Introduction

The diagnostic assessment of the upper airway (UA) in sleep-disordered breathing (SDB), especially for snoring and obstructive sleep apnea (OSA) patients, has undergone tremendous evolution over time. This includes the introduction of several assessment techniques in the awake setting and procedures that can observe the UA vibratory dynamics and/or collapse patterns during sleep.

In 1978, Borowiecki et al. [1] were the first to propose endoscopic evaluation of OSA patients under natural sleep condition. However, this technique was limited to an experimental setting because of the discomfort caused to patients and the long duration needed to assess each patient. Likewise, dynamic imaging techniques (real-time magnetic resonance, computed tomography, etc.) may represent a precise method to study the entire UA simultaneously, but imaging techniques are also very time-consuming and are limited by the high cost for each study. Dynamic UA imaging can be challenging in daily practice as it requires close collaboration between different departments. Furthermore, maneuvers or body positioning during sleep imaging can be almost impossible.

In the last two decades, drug-induced sleep endoscopy (DISE) has gained importance in daily practice because it can take a snapshot of the dynamic, three-dimensional UA during pharmacologically induced sleep, within a short time [2,3,4]. 

In this chapter, we review the literature on the use of DISE in treatment decision-making and analyze the impact of DISE on treatment outcomes.

## 2. DISE Usefulness in Treatment Decision-Making Processes

Recent therapeutic protocols for OSA patients increasingly involve a combined/multimodal surgical and non-surgical therapy, replacing the previous focus on one main treatment option, the so called "one size fits all" approach [5,6,7].

Continuous positive airway pressure (CPAP) remains the first line treatment for patients with moderate to severe OSA. However, CPAP therapy is associated with decreasing compliance over time with reported failure rates ranging from 40 to 85% [8,9,10].

The surgical treatment options for OSA are usually determined by the patient characteristics (UA anatomy, OSA severity, body mass index, and medical comorbidities), surgeon’s experience and preference, and technological equipment availability (e.g., surgical robot, coblator, radiofrequency devices, barbed suture, etc.). In addition, the literature has reported many different types of non-standardized surgical procedures for both palatal and hypopharyngeal levels, performed using a wide variety of techniques with different technological devices [11].

Positional therapy is an effective treatment option for patients with significant positional OSA (POSA) [12]. Furthermore, a mandibular advancement device (MAD) can play an important role in the treatment of selected mild to moderate grades of OSA [13]. Finally, the emerging literature supports the positive effects of myofunctional therapy [14] in OSA patients.

DISE is a first-line UA diagnostic procedure that can help guide treatment option selection for OSA patients. 

DISE allows the visualization of the sites and patterns of partial or total UA collapse under different levels of sedation. This may be useful in the selection of surgical candidates amongst the OSA patients.

Considering the huge variety of surgical options including some non-standard procedures, it is of pivotal importance to diagnose OSA with a sleep study and assess the three-dimensional UA anatomical obstruction using DISE. 

The UA anatomy can change significantly from the awake to the sleep state, mainly due to the decrease in UA musculature tone. Only DISE can visualize the dynamic UA three-dimensional change when the patient is under sedation [15].

According to some authors, DISE may provide useful data to help select the most efficient surgical technique, reduce the rate of unnecessary multilevel (and thus more extensive) surgical resection, and potentially improve surgical success rates [16].

Vanderveken et al. demonstrated the importance of DISE in predicting the success rate of implanted hypoglossal nerve stimulation. The absence of total circumferential palatal collapse during DISE is associated with better therapeutic success [17].

In a retrospective study by Soares et al., it was found that any significant lateral oropharyngeal wall collapse and/or supraglottic collapse during DISE may be associated with worse surgical outcome [18].

More recently, a multicenter cohort study by Green et al. reported that any DISE findings of lateral oropharyngeal wall obstruction and complete tongue obstruction pattern may lead to a higher risk of surgical failure [19]. 

Positional therapy is an effective treatment option for patients with POSA. POSA is defined when the apnea–hypopnea index (AHI) is at least 5 with a supine AHI that is at least 2 times higher than the non-supine AHI [20]. Sleep position must be recorded in a sleep study for the diagnosis of POSA. A sleep study may still miss the diagnosis of POSA if the patient only sleeps in a pure supine or pure non-supine position [21]. Even when the diagnosis of POSA is not made, DISE performed in different sleep positions may still identify potential candidates that may benefit from positional therapy. POSA patients (who may or may not be previously diagnosed) should demonstrate at least partial widening of the upper airway in a non-supine position [21].

Head rotation alone, with the body in supine position, during DISE may be adequate to predict the positive effect of position change on the upper airway size of POSA patients [22], even if the latest literature data highlighted the importance of lateral head and trunk rotation on UA patency during drug-induced sleep endoscopy in POSA patients [23]. Patients with significant improvement in UA patency in a non-supine position will likely benefit from positional therapy.

MAD is a good treatment choice for suitable patients with mild to moderate OSA. To simulate MAD, manual jaw advancement by about 5 mm can be performed during DISE [24]. If good improvement in UA size can be demonstrated with manual jaw advancement during DISE, the patient will likely benefit from MAD therapy [23]. The application of a custom-made simulation-bite in pre-adjusted maximum comfortable protrusion during DISE may further improve the accuracy of the MAD’s effect [25].

Myofunctional therapy has become a new effective therapy for both pediatric and adult OSA. It can be a stand-alone treatment or used in combination with both surgical and/or non-surgical treatment [14]. DISE can be performed before and after myofunctional therapy to assess improvement in the tongue muscular motion during sleep after a duration of therapy. 

## 3. Role of DISE in Predicting Treatment Outcomes

Certal et al. published a recent systematic review comparing UA awake examination versus DISE as diagnostic tools for surgical decision-making. A total of eight studies with 535 patients were included in the review. Surgical treatment was changed after DISE in 50.24% cases. The change in surgical planning was mainly due to the presence of hypopharyngeal or laryngeal dynamic collapse that is only seen on DISE. However, the change in surgical planning did not lead to a significantly higher success rate [26]. 

Very few studies analyzed the actual implications of DISE on surgical success, and the results of these studies appear heterogeneous. Overall, no solid consensus could be reached by analyzing the literature. 

Blumen et al. showed that even when all the obstructed sites on DISE were treated surgically, it did not always lead to treatment success. The additional sites detected by DISE may actually lead to unnecessary procedures. Oversedation, prolonged DISE assessment, oversensitive observation, and misunderstanding of the DISE findings (e.g., missing the primary cause of secondary obstruction sites) may lead to additional artificially obstructed sites [27]. 

In a recent nonrandomized, prospective, multicenter clinical trial, Pang et al. studied 356 OSA patients from nine tertiary clinical centers in seven different countries, including Singapore, Canada, India, Spain, Poland, Israel, and Korea. The authors reported better surgical outcomes for OSA patients with no preoperative DISE (no-DISE group) compared to patients who had undergone DISE. Nevertheless, the authors remarked that the no-DISE group had more nasal surgery performed (*p* < 0.001) which might have led to better outcomes in this group. Furthermore, despite reporting the use of the European Position Paper on DISE protocol, the detailed DISE techniques from different centers were not homogeneous. For example, every center has its own preferred sedative agents, such as intravenous propofol or dexmedetomidine. Finally, remarkably variable surgical techniques were performed by each of the surgeons from the different centers [28].

Eichler et al. also reported significant changes to the final treatment decision from awake to DISE analysis for both surgical options (63.9% of patients) and the MAD patients’ group (78.4%) [29].

Aktas et al. found that the surgical treatment of DISE obstruction sites led to increased treatment success. Their detailed analysis revealed a higher surgical success rate for patients with superior UA obstruction. On the other hand, surgery for inferior UA collapse was associated with a poorer success rate [30].

The addition of real-time cardio-respiratory monitoring during DISE may further improve the correlation between respiratory events and UA collapse [31]. Video recording of DISE may also be useful to study the upper airway patterns in OSA patients.

In conclusion, there is no general consensus on the role of DISE in predicting surgical success [26], even though DISE provides useful information regarding specific UA patterns of collapse (circumferential vs. anteroposterior soft palate pattern of collapse, predominant pharyngeal lateral wall collapse, etc.) that are associated with potential surgical failure.

## 4. Conclusions

The aim of UA assessment in OSA patients is to give a clear insight into the complex pathophysiology of UA collapse and to improve treatment success rates. UA assessment in OSA patients are often limited by the static findings in the awake setting, which usually do not represent the dynamic UA collapse during sleep. DISE provides an alternative method of UA assessment using a fiberoptic endoscope during pharmacologically induced sedation.

It is challenging to systematically review the literature on DISE due to the heterogeneous DISE protocols and highly variable treatment options. So far, the literature has demonstrated that preoperative DISE tends to change the initial surgical planning, but the improvement in surgical outcomes remains an open question. Nevertheless, DISE provides useful UA evaluation, which enables the identification of UA patterns of collapse associated with potential surgical failure.
